# Spatial pattern of distribution of marine invertebrates within a subtidal community: do communities vary more among patches or plots?

**DOI:** 10.1002/ece3.2462

**Published:** 2016-10-22

**Authors:** Chun‐Yi Chang, Dustin J. Marshall

**Affiliations:** ^1^ Centre for Geometric Biology/School of Biological SciencesMonash UniversityClaytonVic.Australia

**Keywords:** community assembly, fouling, scale, sessile, succession, variance partitioning

## Abstract

Making links between ecological processes and the scales at which they operate is an enduring challenge of community ecology. Our understanding of ecological communities cannot advance if we do not distinguish larger scale processes from smaller ones. Variability at small spatial scales can be important because it carries information about biological interactions, which cannot be explained by environmental heterogeneity alone. Marine fouling communities are shaped by both the supply of larvae and competition for resources among colonizers—these two processes operate on distinctly different scales. Here, we demonstrate how fouling community structure varies with spatial scale in a temperate Australian environment, and we identify the spatial scale that captures the most variability. Community structure was quantified with both univariate (species richness and diversity) and multivariate (similarity in species composition) indices. Variation in community structure was unevenly distributed between the spatial scales that we examined. While variation in community structure within patch was usually greater than among patch, variation among patch was always significant. Opportunistic taxa that rely heavily on rapid colonization of free space spread more evenly among patches during early succession. In contrast, taxa that are strong adult competitors but slow colonizers spread more evenly among patches only during late succession. Our findings show significant patchiness can develop in a habitat showing no systematic environmental spatial variation, and this patchiness can be mediated through different biological factors at different spatial scales.

## 1 Introduction

A major goal in ecological research is to identify patterns in biodiversity and understand how these patterns are generated. In community ecology, one of the most important patterns is the variation of communities in space (Godfray & Lawton, 2001; Underwood, Chapman, & Connell, 2000). Choosing the appropriate scale to understand patterns can be difficult, highlighted in ecology as “the problem of scale” (Godfray & Lawton, 2001; Huston, 1999; Levin, 1992). In several early theoretical explorations, MacArthur and co‐workers highlighted the importance of trait–environment relations at the level of local scale (MacArthur & Levins, 1967); however, the importance was dismissed when studying larger regional scale levels (MacArthur & Wilson, 1967). More recent findings suggest that scale dependency is the key to disentangling patterns in community structure (Boulangeat, Gravel, & Thuiller, 2012; Chase & Myers, 2011; Holyoak, Leibold, Mouquet, Holt, & Hoopes, 2005).

The abundance and distribution of organisms can respond to varying factors across scales. For instance, light availability and soil chemistry are known determining factors of terrestrial plant communities at local scales, while precipitation and habitat connectivity are important determining factors at regional scales (Medina et al., 2014; Svenning & Skov, 2005). In bird communities, food‐producing plants determine fruit and nectar availability, which in turn determine both local and regional avifaunal variations (Tellería & Pérez‐Tris, 2003). In marine subtidal habitats, benthic fouling communities are characterized by life histories that are similar to terrestrial plants: sessile adults producing dispersive propagules. In these fouling communities, high levels of variation can often be found on small spatial scales (e.g., from cm to tens of m, Farnsworth & Ellison, 1996; Underwood & Chapman, 1996). These levels of variation could be explained by interactions among different species (i.e., avoidance, facilitation, and predation), or by small‐scale environmental variables (i.e., haphazard scattering of suitable microhabitats, Underwood & Chapman, 1996). As most species of marine fouling communities have a relatively short larval period (in contrast to the seeds of many terrestrial plants), the spatial structure of these communities is thought to be strongly regulated by early colonization and by small‐scale environmental variables (Caley et al., 1996).

**Figure 1:**
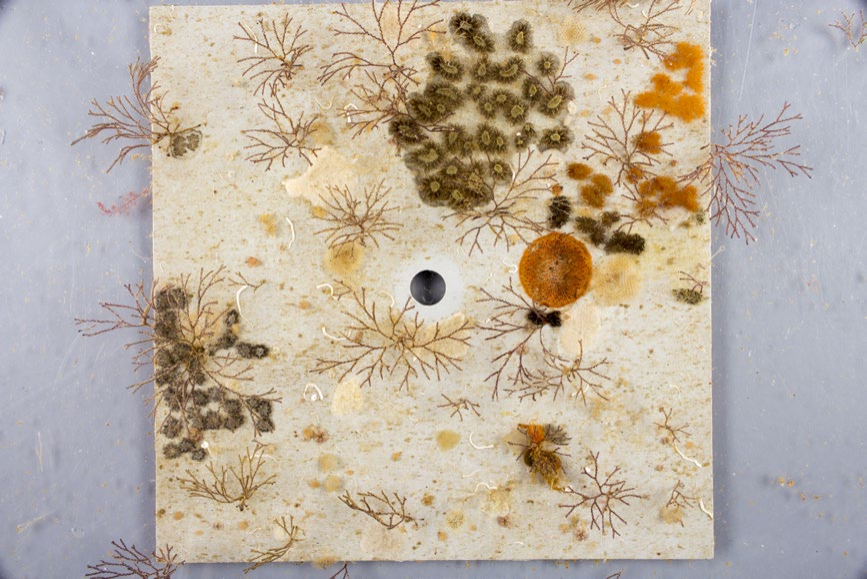
One of the experimental plots bearing intact fouling community. This plot was occupied by many colonies of the arborescent bryozoan *Bugula neritina*, along with other encrusting bryozoans and colonial ascidians. Photograph credit: Chun‐Yi Chang

In establishing the “assembly rules” of communities, a common approach is to explore the relationship between biodiversity and environmental gradients. Such approaches typically focus on *mean* biodiversity changes; much less attention is given to the *variance* around the mean of biodiversity, especially in the absence of obvious environmental differences (but see Benedetti‐Cecchi, 2003; Legendre, Borcard, & Peres‐Neto, 2005; Tuomisto & Ruokolainen, 2006). For instance, beta‐diversity (the difference in species richness among sites) is often quantified along an axis of environmental gradient (e.g., productivity gradient and habitat size gradient) and then regressed against the gradient. The unexplained part (variation in diversity in the absence of environmental differences) is often concluded to be “artificial,” due to uncontrolled factors or pure sampling error (e.g., Olivier & van Aarde, 2014). This artificial view should be updated to reflect recent conceptual synthesis, which emphasizes the relative importance of niche determinism versus neutral stochasticity in community assembly (Chase & Myers, 2011; Vellend et al., 2014). Specifically, the operation of neutral stochasticity has been inferred, when community structural variation is explained by the order of species colonization (De Meester, Vanoverbeke, Kilsdonk, & Urban, 2016), or by habitat spatial structure rather than environmental heterogeneity (Cottenie, 2005). Therefore, residual variance can be as informative as mean diversity changes across gradients, because the residuals carry information about neutral stochasticity. In the case of marine fouling communities, the timing of reproduction, larva settlement choice, and predation may have entirely deterministic cues, but the precise location and timing of these biological processes are purely stochastic relative to each other (Vellend et al., 2014). Therefore, what appears to be unexplained random variation could in fact contain an important ecological signal (Fig. 1).

The challenge of explaining random variation prompted an investigation into the scale dependency of a marine fouling community within a habitat that lacks systematic environmental spatial variation. Hierarchical variance partitioning is a useful way to separate scale‐dependent ecological patterns (Bolker et al., 2009). For instance, adult barnacles compete for planktonic food sources directly with their neighbors within a 0.01–0.1 m range, but produce free‐swimming larvae that disperse and interact with other populations over hundreds of kilometers (Pfeiffer‐Herbert, McManus, Raimondi, Chao, & Chai, 2007). Marine coastal ecosystems are thus hierarchical in structure and should be analyzed as such (e.g., MacNeil et al., 2009).

The goal of this study was to investigate the variation in a marine fouling community on two different spatial scales: a few centimeters apart (plot level) and 10s of m apart (patch level). To achieve this, we took a strictly phenomenological approach in which we had no preconception about what factors might have produced the pattern, except the spatial scale. The variation in the abundance of important functional groups (representing different life‐history strategies), as well as the structural variation of the full community, was the focus of our analysis. Using hierarchical variance partitioning, we separated the variance into different components representing plot and patch scales. Specifically, we asked: Was the total variation in the community mainly due to patch‐level differences? Were the distributions of functional groups (dis)similar across scales?

## 2 Methods

### 2.1 Study site and the fouling community

Experiments were performed within Blairgowrie Marina (38°21′31″S, 144°46′23″E) near the southern tip of Port Phillip Bay, Melbourne, Australia. The marine fouling community of the study region consists of species that compete for space for growth and reproduction (Fig. 1). Competition for food and oxygen can also be intense (Ferguson, White, & Marshall, 2013; Svensson & Marshall, 2015).

From our samples, we isolated three of the major functional groups with distinct growth forms and life‐history strategies, to examine their spatial distribution in detail. Encrusting bryozoans have larvae that are among the first to colonize bare surface during early austral summer. With an encrusting growth form, young colonies take over spaces quickly and become reproductive within 8 weeks. Soon after reproducing, part or all of the adult colonies may begin to senesce, freeing up space for colonization of other species. Encrusting bryozoans represent the functional group with an opportunistic larval stage and larger resource requirements as adults (Hart & Marshall, 2012). Arborescent bryozoans have tree‐like growth forms and are more abundant during mid‐ and late‐assembly stages. With an upright growth form, they exploit resource niches that are different from the other encrusting species, but often eventually displaced from later stage communities. They rely less on being a fast grower and an opportunistic colonizer and represent the functional group with a slower growth rate and weaker competitive abilities. Colonial ascidians represent a competitively dominant functional group with large resource requirements as adults (Russ, 1982; Sams & Keough, 2012b). They are less competitive as juveniles and can alternate between different life‐history strategies depending on the surrounding biotic and abiotic environment (Stoner, 1992).

### 2.2 Experiment setup

Underwater patches were created using PVC panels (55 × 55 cm) for fouling communities to establish. There were 16 plots per patch; each plot was 11 × 11 cm. Each patch carried 16 plots which were placed 2 cm apart from one another. Care was taken so that plots of the same patch remained independent from each other: Every 30 days during sampling, plots were changed to a different location within their patch, determined by a random number generator (R Development Core Team, 2011). The surfaces of plots were roughened with sandpaper to encourage larvae settlement. Patches were hung horizontally on a floating pontoon, submerged 1 m below the water surface facing downwards. The floating pontoon was 3 m wide, 100 m long. The water depth ranged from 5.5 to 6 m.

### 2.3 Field program

To quantify the spatial variation, we monitored 12 patches over a period of 3 months. We sampled each patch every 30 days during the austral summer (from December 2012 to the end of February 2013). During sampling, four randomly chosen plots, determined by a random number generator (R Development Core Team, 2011), from each patch were brought back to the laboratory and photographed. The abundance of a species in a plot was quantified in terms of its percentage cover: the area occupied by a species divided by total plot area. A subsampling method was used to generate the percentage cover estimate for all species: Computer software (Coral Point Count with Excel extensions, Kohler & Gill, 2006) was used to generate 100 randomly distributed points over each photograph. All visible, sessile species under the points were recorded. Photographic sampling is a common practice in quantifying the percentage cover of fouling communities (e.g., Drummond & Connell, 2005). We use “species cover” hereafter to refer to the multivariate species composition data that characterized each plot.

### 2.4 Modeling population and community structure variation

The approach we took was a phenomenological one, that is, in our experimental design, we did not include any predictor variable assumed to relate to a particular mechanism. The only “treatment” to the community was the spatial nestedness of plots into patches, which was modeled as a random factor. Here, the key assumption is that the environment showed no systematic spatial variation—obviously, no natural habitat is perfectly homogeneous. Homogeneity of environmental variables depends on the scale of investigation which, in our study, was the scale of the pontoon. We assumed that the environment did not vary systematically along the pontoon. If such environmental gradient exists, assigning patch as a random factor would be invalid, as it would instead represent an environmental gradient. We believe our assumption was justified for the following two reasons. First, the artificial substrata effectively eliminated any topological differences that may influence initial larval settlement choice. Second, species cover in any given patch only showed random deviations from the sitewide pattern, with no evidence of directional, linear change along the pontoon (see Fig. S1 for supporting data). Therefore, we can proceed to investigate the plot‐ and patch‐level variation, which was independent of where the patch was located on the pontoon.

To quantify population and community variation at the patch level and at the plot level, and to investigate whether they varied differently across two scales, we analyzed the spatial distribution of selected taxa that represent dominant growth forms (encrusting bryozoans, arborescent bryozoans, and colonial ascidians). For community structure, we calculated three univariate summary statistics from the multivariate species cover data: species richness (*S*), loadings from correspondence analysis (CA) axis 1, and loadings from CA axis 2 (Oksanen et al., 2016). CA can be used to summarize community structure when species abundances are quantified in terms of frequencies (the number of points in our case). CA preserves the chi‐square distances among sampling units, which ensures that the influence of rare species is not overlooked. Population abundance distribution and community summary statistics were then modeled using a hierarchical random‐effects model,

$$y=\mu +\text{Patch}∼N(0,\sigma^{2})+\varepsilon$$

fitted using restricted maximum likelihood (MIXED procedure of SAS 9.4, SAS Institute, Cary, NC). *y* is the response variable; μ is the overall intercept; Patch is a categorical variable representing patch identity, which accounted for the patch‐level variance and was modeled as a random effect. The model had no fixed term and estimated the patch effects based on the random deviations (normally distributed with mean 0, variance σ^2^) of summary statistics from the fixed patch means. ε is the error term that accounted for the plot‐level variance. The total variance was partitioned into those due to patches and those due to plots within patches (the error term). Significance tests were performed using likelihood ratio tests. Our main goal was to partition the spatial variation; therefore, monthly samples were analyzed separately.

## 3 Results

Throughout the sampling period, we recorded 33 taxa in the fouling community, of which 31 were invertebrates and two were algae. Most community members could be identified to the species level; for those that could not be identified to species using a digital photograph, morphospecies were assigned based on their morphological features (a list of common taxa along with their adult growth form can be found in Table S1).

### 3.1 Abundance and distribution of dominant taxa

**Figure 2:**
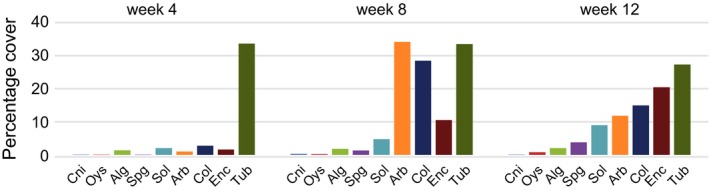
Percentage cover of nine most abundant taxa found at our field site. Data represent cover of each taxon summed across all plots. Cni–cnidarians; Oys–oyster; Alg–algae; Spg–sponges; Sol–solitary ascidians; Arb–arborescent bryozoans; Col–colonial ascidians; Enc–encrusting bryozoans; Tub–tubeworms. Refer to Table S1 for growth form of each taxon

We saw different taxa dominating the community at different sampling times (Fig. 2). Tubeworms covered *c*. 30% of the space at weeks 4 and 8 and were overgrown by other organisms at week 12. Solitary ascidians (that include three morphospecies) and encrusting bryozoans (that include seven species) increased steadily in their space cover over time. At week 12, encrusting bryozoans occupied *c*. 20% of the space while solitary ascidians had *c*. 10% of space cover. Arborescent bryozoans (includes four species) and colonial ascidians had their maximum cover only at week 8 (34% and 28%, respectively) and had less cover at weeks 4 and 12.

**Figure 3:**
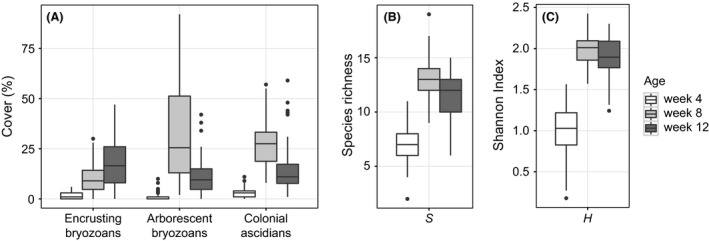
Plot‐to‐plot variation in abundance of encrusting bryozoans, arborescent bryozoans, and colonial ascidians (A). Plot‐to‐plot variation in species richness *S* (B). Plot‐to‐plot variation in Shannon diversity *H* (C). Observations at the plot level are presented, and thus, the size of the boxes represents plot‐level variation. Thick lines in boxes represent medians; lower and upper hinges of boxes correspond to first and third quartiles of the observation; lower and upper whiskers span through the 3/2 interquartile range; observations beyond the end of the whiskers are outliers (solid dots)

Apart from these overall patterns, for the three important functional groups, we further examined their abundance variation from plot to plot. Across different groups, plot‐level variance always fluctuated in response to the mean (Fig. 3A). Higher variance was always associated with a higher mean. Consequently, spatial variability of encrusting bryozoans increased in time with its overall percent cover, with the greatest variation observed at week 12. In contrast, arborescent bryozoans and colonial ascidians both showed the highest amount of plot‐to‐plot variation during mid‐succession, at week 8.

### 3.2 Patterns in community structure

Similar to the patterns of functional groups, community structure also varied considerably in space (Fig. 3B, C). At week 4, we recorded 22 taxa sitewide (species richness *S *=* *22). The average *S* in any given plot was 7.5. At week 8, sitewide *S* increased to 28, with an average *S* for a given plot being 13. At week 12, both sitewide and plot‐averaged *S* decreased slightly to become 21 and 12.5, respectively. Shannon diversity index *H* showed a temporal pattern similar to *S* where the community was most diverse during mid‐succession (Fig. 3C). Both *S* and *H* had similar amount of spatial variability over time as the size of each box was similar over time. Nonetheless, the *H* observed in each plot at week 4 appeared to tend toward smaller values, suggesting that plots at this stage were mostly colonized by only a few common taxa.

### 3.3 Patch‐level versus plot‐level variability

**Figure 4:**
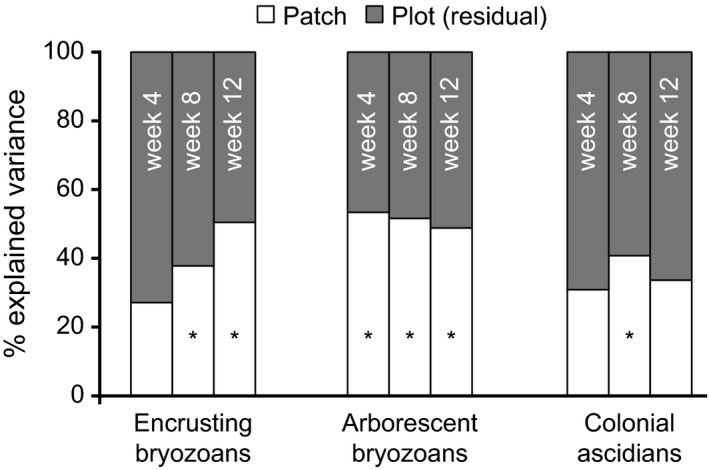
Plot‐to‐plot variation in abundance of encrusting bryozoans, arborescent bryozoans, and colonial ascidians partitioned into those explained at the patch level, and those explained at the level of plots within patches (residual). An asterisk indicates that the model with patch information explained the abundance distribution significantly better than the null model (one without patch information) at a significance level of *p *=* *.05

Results of the variance partitioning showed that patch differences were sometimes important in maintaining the spatial variation in the abundance distribution of the three functional groups, depending on the identity of the functional group (Fig. 4). For encrusting bryozoans, the observed plot‐to‐plot variance in their abundance was not significant at the patch scale at week 4 (early succession). Models with and without patch identity as a random factor fitted the encrusting bryozoan data equally well under our likelihood ratio tests. This suggests that, during early succession, the abundance of encrusting bryozoans found in any two plots did not depend on whether or not they were in the same patch. At weeks 8 and 12, the model with patch identity as a random factor performed significantly better in explaining the encrusting bryozoan distribution than the model without patch identity.

For arborescent bryozoans (Fig. 4), the model with patch random effect always had a better fit than the model ignoring patch identity, regardless of the timing of sampling. In other words, arborescent bryozoans always had significant variation both at the level of patch and plot.

For populations of colonial ascidians (Fig. 4), aggregation at the patch scale was only significant at week 8 (mid‐succession). During early and late succession, their spatial distribution did not show an obvious pattern following patch identity.

**Figure 5:**
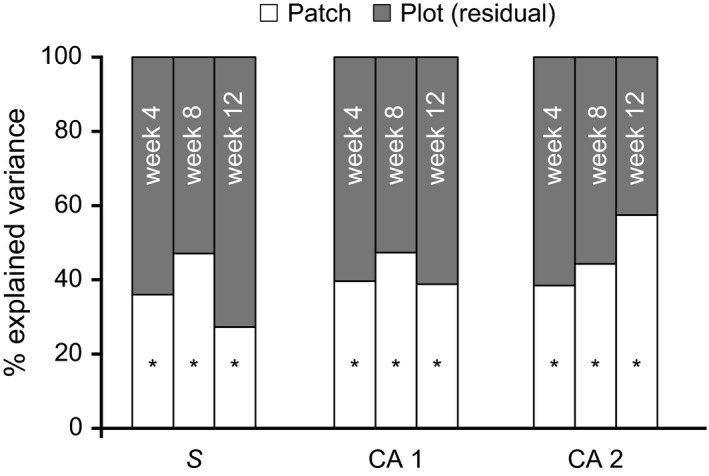
Plot‐to‐plot variation in community structure partitioned into those explained at the patch level, and those explained at the level of plots within patches (residual). Community structure is quantified in three ways: species richness (*S*) and the first two axes of correspondence analysis (CA). An asterisk indicates that the model with patch information explained the distribution significantly better than the null model (one without patch information) at a significance level of *p *=* *.05

For the variation in community structure, the patch‐scale pattern was always significant (Fig. 5), regardless of how community structure was quantified. This suggests that we could always expect two plots to develop more similar communities if they were in the same patch. By further calculating all pairwise plot similarity and separating them into among‐ and within‐patch pairs, we found that communities in different patches were on average 18% more different than communities developed in the same patch (Fig. S2). Among‐patch similarities were always significantly lower than within‐patch similarities.

## 4 Discussion

Individual populations as well as community structure showed pronounced variation among different plots at our field site. We also found that patch contributed significantly to this Plot‐to‐plot pattern. That is, when plots were grouped according to their patch identity, patch‐to‐patch variation emerged. For the distribution of important functional groups, the importance of patch effects differed across different stages of community assembly; nonetheless, patch effects were always a significant component contributing to the variation of the overall community. In the current study, distance between patches ranged from 3 m up to 80 m. At this spatial scale, both populations and communities have been reported to exhibit high spatial variability (Coleman, 2002; Underwood & Chapman, 1996). In subtidal habitats, variability at this spatial scale can be generated by environmental heterogeneity, which can in turn facilitate differences in the abundance of habitat‐engineering species, nutrient turnover, or larvae dispersal (Kendrick & Walker, 1995; Sams & Keough, 2013b). With our experimental setup, systematic environmental variation was minimized by two key elements. First, all patches were located on a single pontoon inside a marina that had particularly low wave energy throughout. Second, we homogenized substrata topology and orientation for all experiments using suspended PVC panels. We therefore suggest that processes other than systematic environmental variation were the principal drivers behind patch‐scale variation. For instance, the dispersal of larvae can introduce variability at both large and small spatial scales. While large‐scale larval dispersal can be understood through the study of regional ocean current dynamics, small‐scale patterns of larval dispersal are often driven by biological factors (Caro, Navarrete, & Castilla, 2010). Here, the length of larval period, larvae swimming behavior, and settlement choice could all determine small‐scale dispersal patterns.

For whole‐community variation, patch effects were always significant for weeks 4, 8, and 12 assemblages, suggesting that initial dispersal alone did not generate the patch effects. Biotic interactions that took place after settlement and recruitment may have promoted subsequent patch‐scale differences. Accumulating evidence now suggests that the order of species arrival can have a great influence on subsequent assemblage structure (Chang & Marshall, 2016; Vellend et al., 2014). By arriving early into new patches, weak resource competitors could avoid extinction in a community through niche preemption (this is particularly noticeable in arborescent species). Similarly, dominant species could also attain monopolization of individual plots by arriving earlier than other members from the same guild (De Meester et al., 2016; Keough, 1984). Both negative (competitive) and positive (facilitative) interactions between organisms would certainly have generated and maintained the community variation at the patch scale after week 8, where the three important functional groups occupied *c*. 70% of plot surface. As populations increased in their percentage cover, colonies overgrowing each other were more commonly observed (authors’ personal observation). Furthermore, the outcome of biotic interactions may also depend on trait differences among taxa, which would in turn affect the overall community pattern. Trade‐offs between life‐history traits are an important mechanism that maintain diversity in communities (Tilman, 2004). A classic example among fouling communities is the colonization‐competition trade‐off (Edwards & Stachowicz, 2010; Sousa, 1979), in which different species invest differently in the reproduction versus vegetative growth. If species rely heavily on the larval period, they may have traits such as better larval dispersal ability or rapid sexual maturity. Other species investing in postrecruitment stages may grow slower, but may eventually become better competitors for resources (e.g., space). The arborescent bryozoans appear to adopt the former strategy as we observed an early pulse of settlers that distributed relatively evenly over the study site, and then quickly occupied over 30% of the plots in less than 6 weeks. In contrast, solitary ascidians such as *Pyura* may adopt the latter strategy, as they were hardly found during the first 4 weeks in our plots, but gradually overgrew other species as succession occurred. Dalby (1995) reported that *Pyura* colonize steadily throughout the year but rarely dominate settlement events. Such segregation in phenology may be an important way in which diversity is maintained (Butler & Chesson, 1990).

Another important factor driving different patterns among different populations is the asymmetric interaction strength among species. A competitive dominant may inhibit the growth of one species while facilitate the other. Furthermore, ontogeny may change how species interact throughout life stages. The combined net effect of asymmetric interactions may thus generate different community patterns at different ages. The larvae of some species with high space demand have been shown to avoid settling near large space‐occupying competitors (Gaines & Roughgarden, 1985; Grosberg, 1981), such as *Pyura* in our study system. Meanwhile, in other studies, *Pyura* have also been shown to facilitate adjacent bryozoan larval settlement and colony growth (Sams & Keough, 2013a). Moreover, depending on resource availability, interactions within taxa may propagate to the community level with completely opposite effects. For instance, if the patch is already crowded, then the facilitated *Bugula* spp. settlement by *Pyura* may not last long enough to affect succession at later stages because *Bugula* are short‐lived. However, if there is plenty of free space, *Bugula* spp. have the potential to alter community structure through direct interaction with colonizers of longer‐lived species (Menge, 1995).

Similar to the community‐level patterns, percentage cover of three functional groups showed significant spatial variation at the patch scale. The magnitude of fluctuation in populations can be much greater than those community‐wide diversity patterns (Fig. 3). There are other systems known to have similar contrasts (community vs. population variability). Tilman (1996) reported that variations in grassland total community biomass were relatively stable compared with population abundance variation. Our results demonstrate that the difference in variation between populations and communities can be observed on shorter, within‐generation time scales. Whether the same set of mechanisms are in operation between long and short time scales is not clear at this point. In our observations, there seems to be complementary space occupiers. For instance, when arborescent bryozoans failed to establish in a plot, colonial ascidians such as *Botrylloides* or *Didemnum* were very likely to take advantage of the free space. Thus, so long as this complementary relationship holds among species, there will be relatively little variation in terms of species richness. However, the coarse taxonomic resolution used in our study should be interpreted with caution. Major colonial ascidian populations were lumped into functional groups because species identification was problematic with our photographic sampling. Therefore, it is possible that our analysis underestimates the total community variance if populations varied more at the species level than at other coarser levels. Nevertheless, it is clear that spatial variation among patches was significant at both population and community levels employed in the current study.

Initial recruits of encrusting bryozoans (week 4) showed no significant patterns at the patch scale. Patch effects only occurred at weeks 8 and 12. This suggests that the initial distribution of encrusting bryozoans was largely random at both plot and patch scales that we explored. Patch effects that emerged later were most likely due to the higher amount of space occupied by this functional group and the resulting greater biotic interactions among community members. A dominant encrusting bryozoan species, *Watersipora subtorquata*, was found to suffer from higher mortality when they were adjacent to barnacles and ascidians (Sams, Warren‐Myers, & Keough, 2015). In accordance with our results, this disadvantage in survival only became apparent 8 weeks after settlement.

The distribution of arborescent bryozoans showed significant patch effects throughout the experiment. Arborescent bryozoans are generally weak competitors and their adults are known to facilitate the recruitment of tubeworms (Osman & Whitlatch, 1995; Sams & Keough, 2012a), both implying that arborescent bryozoans may be susceptible to strong biotic interactions regardless of their developmental stage, which is supported by our observation.

Colonial ascidians only showed significant patch effects at week 8, while at week 4 and 12, their distribution was mostly random at both the plot and patch scales. The most abundant colonial ascidian, *Didemnum*, was a dominant competitor and was capable of overgrowing other species and monopolizing an entire plot in some of our experiments. Their overgrowth regardless of the presence of other neighbors could explain their distribution at week 12. Despite this, our results at week 4 are not consistent with prediction from previous studies that recruits of *Didemnum* would experience strong biotic interactions when young and therefore should exhibit patchy distributions (Sams & Keough, 2012a). *Didemnum* recruits can alternate between different life‐history strategies, which would result in high phenotypic (trait) plasticity. Consequently, colonial ascidian populations at week 4 may show significant patch effects if we examined their trait values instead of percentage cover.

Overall, we find that communities exhibit significant variation among patches and within patches. Generally, communities in different patches are less similar to each other than communities within the same patch. Stochastic settlement seems to drive early variation in communities among patches, but competitive interactions among species appear to dominate later. Opportunistic taxa that rely heavily on fast colonization of free space showed random distribution at both scales, but only during early assembly when biotic interactions were the weakest. Slow colonizers that develop to become strong competitors as adults tend to show random distribution during later assembly stages. Nevertheless, we find no evidence for communities among patches to become more similar over time.

## Conflict of Interest

None declared.

## Funding Information

CYC is supported by Australian International Postgraduate Research Scholarship and Taiwanese Ministry of Education Scholarship. DJM is supported by the Australian Research Council.

## References

[ece32462-bib-0001] Benedetti‐Cecchi, L. (2003). The importance of the variance around the mean effect size of ecological processes. Ecology, 84, 2335–2346.

[ece32462-bib-0002] Bolker, B. M. , Brooks, M. E. , Clark, C. J. , Geange, S. W. , Poulsen, J. R. , Stevens, M. H. H. , & White, J.‐S. S. (2009). Generalized linear mixed models: A practical guide for ecology and evolution. Trends in Ecology & Evolution, 24, 127–135. 19185386 10.1016/j.tree.2008.10.008

[ece32462-bib-0003] Boulangeat, I. , Gravel, D. , & Thuiller, W. (2012). Accounting for dispersal and biotic interactions to disentangle the drivers of species distributions and their abundances. Ecology Letters, 15, 584–593. 22462813 10.1111/j.1461-0248.2012.01772.x PMC3999639

[ece32462-bib-0004] Butler, A. J. , & Chesson, P. L. (1990). Ecology of sessile animals on sublittoral hard substrata: The need to measure variation. Australian Journal of Ecology, 15, 521–531.

[ece32462-bib-0005] Caley, M. J. , Carr, M. H. , Hixon, M. A. , Hughes, T. P. , Jones, G. P. , & Menge, B. A. (1996). Recruitment and the local dynamics of open marine populations. Annual Review of Ecology and Systematics, 27, 477–500.

[ece32462-bib-0006] Caro, A. U. , Navarrete, S. A. , & Castilla, J. C. (2010). Ecological convergence in a rocky intertidal shore metacommunity despite high spatial variability in recruitment regimes. Proceedings of the National Academy of Sciences USA, 107, 18528–18532. 10.1073/pnas.1007077107 PMC2972975 20937867

[ece32462-bib-0007] Chang, C.‐Y. , & Marshall, D. J. (2016). Quantifying the role of colonization history and biotic interactions in shaping communities—A community transplant approach. Oikos, doi: 10.1111/oik.03478. In press.

[ece32462-bib-0008] Chase, J. M. , & Myers, J. A. (2011). Disentangling the importance of ecological niches from stochastic processes across scales. Philosophical Transactions of the Royal Society of London B: Biological Sciences, 366, 2351–2363. 21768151 10.1098/rstb.2011.0063 PMC3130433

[ece32462-bib-0009] Coleman, M. A. (2002). Small‐scale spatial variability in intertidal and subtidal turfing algal assemblages and the temporal generality of these patterns. Journal of Experimental Marine Biology and Ecology, 267, 53–74.

[ece32462-bib-0010] Cottenie, K. (2005). Integrating environmental and spatial processes in ecological community dynamics. Ecology Letters, 8, 1175–1182. 21352441 10.1111/j.1461-0248.2005.00820.x

[ece32462-bib-0011] Dalby, J. E. (1995). Consequences of aggregated living in the ascidian *Pyura stolonifera*: Evidence for non‐contact intraspecific competition. Marine and Freshwater Research, 46, 1195–1199.

[ece32462-bib-0012] De Meester, L. , Vanoverbeke, J. , Kilsdonk, L. J. , & Urban, M. C. (2016). Evolving perspectives on monopolization and priority effects. Trends in Ecology & Evolution, 31, 136–146. 26778169 10.1016/j.tree.2015.12.009

[ece32462-bib-0013] Drummond, S. P. , & Connell, S. D. (2005). Quantifying percentage cover of subtidal organisms on rocky coasts: A comparison of the costs and benefits of standard methods. Marine and Freshwater Research, 56, 865–876.

[ece32462-bib-0014] Edwards, K. F. , & Stachowicz, J. J. (2010). Multivariate trade‐offs, succession, and phenological differentiation in a guild of colonial invertebrates. Ecology, 91, 3146–3152. 21141176 10.1890/10-0440.1

[ece32462-bib-0015] Farnsworth, E. J. , & Ellison, A. M. (1996). Scale‐dependent spatial and temporal variability in biogeography of mangrove root epibiont communities. Ecological Monographs, 66, 45–66.

[ece32462-bib-0016] Ferguson, N. , White, C. R. , & Marshall, D. J. (2013). Competition in benthic marine invertebrates: The unrecognized role of exploitative competition for oxygen. Ecology, 94, 126–135. 23600247 10.1890/12-0795.1

[ece32462-bib-0017] Gaines, S. , & Roughgarden, J. (1985). Larval settlement rate: A leading determinant of structure in an ecological community of the marine intertidal zone. Proceedings of the National Academy of Sciences USA, 82, 3707–3711. 10.1073/pnas.82.11.3707 PMC397856 16593571

[ece32462-bib-0018] Godfray, H. C. J. , & Lawton, J. H. (2001). Scale and species numbers. Trends in Ecology & Evolution, 16, 400–404. 11403873 10.1016/s0169-5347(01)02150-4

[ece32462-bib-0019] Grosberg, R. K. (1981). Competitive ability influences habitat choice in marine invertebrates. Nature, 290, 700–702.

[ece32462-bib-0020] Hart, S. P. , & Marshall, D. J. (2012). Advantages and disadvantages of interference‐competitive ability and resource‐use efficiency when invading established communities. Oikos, 121, 396–402.

[ece32462-bib-0021] Holyoak, M. , Leibold, M. A. , Mouquet, N. , Holt, R. D. , & Hoopes, M. F. (2005). Metacommunities—A framework for large‐scale community ecology In Holyoak M., Leibold M. A., & Holt R. D. (Eds.), Metacommunities: Spatial dynamics and ecological communities (pp. 1–31). Chicago, IL: The University of Chicago Press.

[ece32462-bib-0022] Huston, M. A. (1999). Local processes and regional patterns: Appropriate scales for understanding variation in the diversity of plants and animals. Oikos, 86, 393–401.

[ece32462-bib-0023] Kendrick, G. A. , & Walker, D. I. (1995). Dispersal of propagules of *Sargassum* spp. (Sargassaceae: Phaeophyta): Observations of local patterns of dispersal and consequences for recruitment and population structure. Journal of Experimental Marine Biology and Ecology, 192, 273–288.

[ece32462-bib-0024] Keough, M. J. (1984). Dynamics of the epifauna of the bivalve pinna bicolor: Interactions among recruitment, predation, and competition. Ecology, 65, 678–688.

[ece32462-bib-0025] Kohler, K. E. , & Gill, S. M. (2006). Coral Point Count with Excel extensions (CPCe): A Visual Basic program for the determination of coral and substrate coverage using random point count methodology. Computers & Geosciences, 32, 1259–1269.

[ece32462-bib-0026] Legendre, P. , Borcard, D. , & Peres‐Neto, P. R. (2005). Analyzing beta diversity: Partitioning the spatial variation of community composition data. Ecological Monographs, 75, 435–450.

[ece32462-bib-0027] Levin, S. A. (1992). The problem of pattern and scale in ecology. Ecology, 73, 1943–1967.

[ece32462-bib-0028] MacArthur, R. , & Levins, R. (1967). The limiting similarity, convergence, and divergence of coexisting species. The American Naturalist, 101, 385.

[ece32462-bib-0029] MacArthur, R. H. , & Wilson, E. O. (1967). The theory of island biogeography. Princeton, NJ: Princeton University Press.

[ece32462-bib-0030] MacNeil, M. A. , Graham, N. A. J. , Polunin, N. V. C. , Kulbicki, M. , Galzin, R. , Harmelin‐Vivien, M. , & Rushton, S. P. (2009). Hierarchical drivers of reef‐fish metacommunity structure. Ecology, 90, 252–264. 19294930 10.1890/07-0487.1

[ece32462-bib-0031] Medina, N. G. , Albertos, B. , Lara, F. , Mazimpaka, V. , Garilleti, R. , Draper, D. , & Hortal, J. (2014). Species richness of epiphytic bryophytes: Drivers across scales on the edge of the Mediterranean. Ecography, 37, 80–93.

[ece32462-bib-0032] Menge, B. A. (1995). Indirect effects in marine rocky intertidal interaction webs: Patterns and importance. Ecological Monographs, 65, 21–74.

[ece32462-bib-0033] Oksanen, J. , Blanchet, F. G. , Kindt, R. , Legendre, P. , Minchin, P. R. , O'Hara, R. B. , … Wagner, H. (2016). vegan: Community ecology package. R package version 2.3‐3. Available at: https://cran.rproject.org/web/packages/vegan/ (Accessed 5 september 2016).

[ece32462-bib-0034] Olivier, P. I. , & van Aarde, R. J. (2014). Multi‐scale sampling boosts inferences from beta diversity patterns in coastal forests of South Africa. Journal of Biogeography, 41, 1428–1439.

[ece32462-bib-0035] Osman, R. W. , & Whitlatch, R. B. (1995). The influence of resident adults on recruitment: A comparison to settlement. Journal of Experimental Marine Biology and Ecology, 190, 169–198.

[ece32462-bib-0036] Pfeiffer‐Herbert, A. S. , McManus, M. A. , Raimondi, P. T. , Chao, Y. , & Chai, F. (2007). Dispersal of barnacle larvae along the central California coast: A modeling study. Limnology and Oceanography, 52, 1559–1569.

[ece32462-bib-0037] R Development Core Team (2011). R: A language and environment for statistical computing. Vienna, Austria: R Foundation for Statistical Computing.

[ece32462-bib-0038] Russ, G. R. (1982). Overgrowth in a marine epifaumal community: Competitive hierarchies and competitive networks. Oecologia, 53, 12–19. 10.1007/BF00377130 28310597

[ece32462-bib-0039] Sams, M. A. , & Keough, M. J. (2012a). Contrasting effects of variable species recruitment on marine sessile communities. Ecology, 93, 1153–1163. 22764501 10.1890/11-1390.1

[ece32462-bib-0040] Sams, M. A. , & Keough, M. J. (2012b). Effects of pulse versus steady recruitment on sessile marine communities. Oecologia, 170, 209–219. 22392762 10.1007/s00442-012-2284-1

[ece32462-bib-0041] Sams, M. A. , & Keough, M. J. (2013a). Early recruitment variation and an established dominant alter the composition of a temperate fouling community. Marine Ecology Progress Series, 486, 79–91.

[ece32462-bib-0042] Sams, M. A. , & Keough, M. J. (2013b). Effects of early recruits on temperate sessile marine community composition depend on other species recruiting at the time. Oecologia, 173, 259–268. 23386046 10.1007/s00442-013-2597-8

[ece32462-bib-0043] Sams, M. A. , Warren‐Myers, F. , & Keough, M. J. (2015). Increased larval planktonic duration and post‐recruitment competition influence survival and growth of the bryozoan *Watersipora subtorquata* . Marine Ecology Progress Series, 531, 179–191.

[ece32462-bib-0044] Sousa, W. P. (1979). Disturbance in marine intertidal boulder fields: The nonequilibrium maintenance of species diversity. Ecology, 60, 1225–1239.

[ece32462-bib-0045] Stoner, D. S. (1992). Vertical distribution of a colonial ascidian on a coral reef: The roles of larval dispersal and life‐history variation. The American Naturalist, 139, 802–824.

[ece32462-bib-0046] Svenning, J.‐C. , & Skov, F. (2005). The relative roles of environment and history as controls of tree species composition and richness in Europe. Journal of Biogeography, 32, 1019–1033.

[ece32462-bib-0047] Svensson, J. R. , & Marshall, D. J. (2015). Limiting resources in sessile systems: Food enhances diversity and growth of suspension feeders despite available space. Ecology, 96, 819–827. 26236877 10.1890/14-0665.1

[ece32462-bib-0048] Tellería, J. L. , & Pérez‐Tris, J. (2003). Seasonal distribution of a migratory bird: Effects of local and regional resource tracking. Journal of Biogeography, 30, 1583–1591.

[ece32462-bib-0049] Tilman, D. (1996). Biodiversity: Population versus ecosystem stability. Ecology, 77, 350–363.

[ece32462-bib-0050] Tilman, D. (2004). Niche tradeoffs, neutrality, and community structure: A stochastic theory of resource competition, invasion, and community assembly. Proceedings of the National Academy of Sciences USA, 101, 10854–10861. 10.1073/pnas.0403458101 PMC503710 15243158

[ece32462-bib-0051] Tuomisto, H. , & Ruokolainen, K. (2006). Analyzing or explaining beta diversity? Understanding the targets of different methods of analysis. Ecology, 87, 2697–2708. 17168014 10.1890/0012-9658(2006)87[2697:aoebdu]2.0.co;2

[ece32462-bib-0052] Underwood, A. J. , & Chapman, M. G. (1996). Scales of spatial patterns of distribution of intertidal invertebrates. Oecologia, 107, 212–224. 10.1007/BF00327905 28307307

[ece32462-bib-0053] Underwood, A. J. , Chapman, M. G. , & Connell, S. D. (2000). Observations in ecology: You can't make progress on processes without understanding the patterns. Journal of Experimental Marine Biology and Ecology, 250, 97–115. 10969165 10.1016/s0022-0981(00)00181-7

[ece32462-bib-0054] Vellend, M. , Srivastava, D. S. , Anderson, K. M. , Brown, C. D. , Jankowski, J. E. , Kleynhans, E. J. , … Xue, X. (2014). Assessing the relative importance of neutral stochasticity in ecological communities. Oikos, 123, 1420–1430.

