# Supplementary material for: Spatial pattern of distribution of marine invertebrates within a subtidal community: do communities vary more among patches or plots?
Source: Ecol Evol. 2016 Oct 22;6(22):8330–7. doi: 10.1002/ece3.2462 (PMC5108281; doi:10.1002/ece3.2462)
Supplement: Supplementary file 1 [file ECE3-6-8330-s001.docx]

**Ecology and Evolution**

**Supplementary materials**

**Spatial pattern of distribution of marine invertebrates within a subtidal community: do communities vary more among patches or plots?**

Chun-Yi Chang [changcy.mlml@gmail.com](mailto:changcy.mlml@gmail.com)

Dustin J. Marshall [dustin.marshall@monash.edu](mailto:dustin.marshall@monash.edu)

Centre for Geometric Biology/School of Biological Sciences, Monash University, Victoria, Australia, 3800


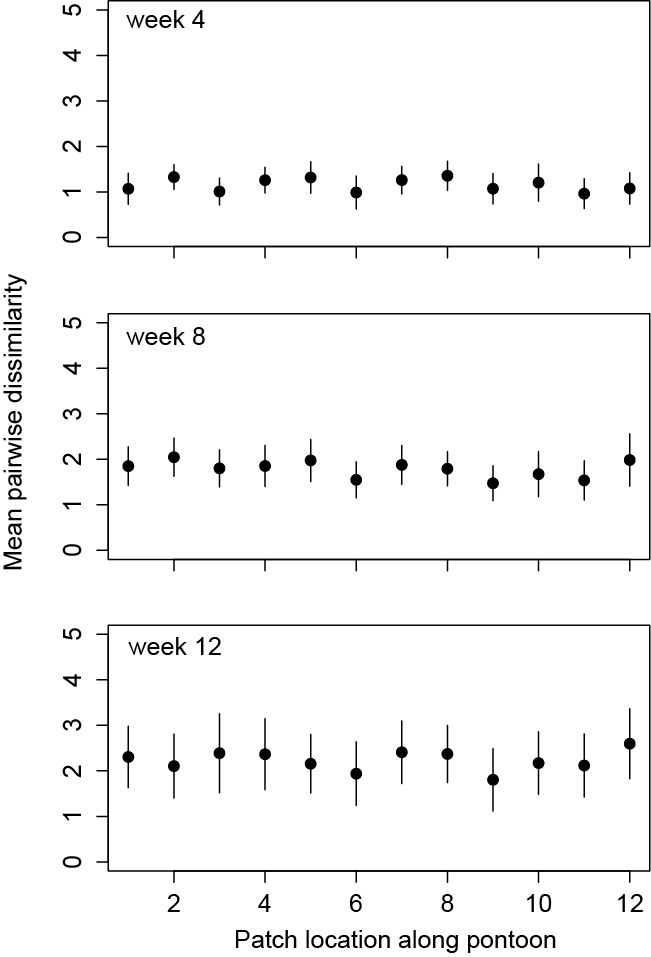


Figure S1. Multivariate dissimilarity in community structure among patches. This was obtained by calculating all pair-wise dissimilarities among plots (from the multivariate species cover data) and then taking the patch average. Take patch 1 for instance (the first solid circle to the left), the dissimilarity value quantifies how different patch 1 was in relative to all other patches. The purpose was to show systematic variation in community structure along the pontoon, if any existed. A directional change in dissimilarity along the pontoon, either with increasing or decreasing similarity, would indicate patch-to-patch community structure changed predictably following some environmental gradients. We chose to look for linear gradient along the pontoon because, based on our observation of the marina, our only reasonable hypothesis was that the end of the pontoon might have been exposed to a different environmental condition than other parts of the pontoon. The cause of this difference may be wave exposure since the end received less protection from the breakwater (pontoon ends near the entrance of the marina). Our results from week 4 to 12 show random deviations from the site average and no such directional change. This indicates no obvious assemblage change along the pontoon, which in turn suggests that environmental gradients was lacking. χ^2^ distance was used as the dissimilarity index. Bars represent ±1 standard deviation.

Table S1. List of common taxa found in our fouling community. Commonness is defined as having a percent cover equal to or more than 2% in at least one patch. The growth form of each species is also described: A-arborescent; E-encrusting; C-colonial; S-solitary.

| Taxa | Growth form |
| --- | --- |
| MACROALGAE |  |
| *Bryopsis plumosa* | A |
| *Asparagopsis armata* | A |
|  |  |
| ANNELIDA |  |
| *Hydroides elegans* | S |
| *Pomatocerus taeniata* | S |
| *Spirorbis spirorbis* | S |
|  |  |
| BRYOZOA |  |
| *Bugula dentata* | AC |
| *Bugula stolonifera* | AC |
| *Bugula flabellata* | AC |
| *Bugula neritina* | AC |
| *Schizoporella spp.* | EC |
| *Membranipora membranacea* | EC |
| *Watersipora subtorquata* | EC |
| *Celleporella hyalina* | EC |
| *Microporella ciliata* | EC |
|  |  |
| CNIDARIA |  |
| *Hydroidae sp.* | A |
|  |  |
| ASCIDIAN |  |
| *Ascidiella aspersa* | S |
| *Botrylloides leachi* | EC |
| *Botryllus schlosseri* | EC |
| *Didemnum sp.* | EC |
| *Ciona intestinalis* | S |
| *Pyura stolinifera* | S |
| *Diplosoma listernium* | EC |
|  |  |
| PORIFERA |  |
| *Sycon ciliatum* | AS |


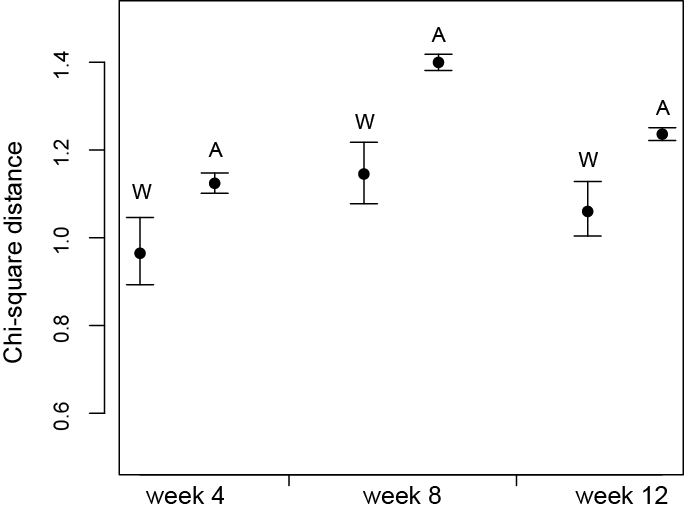


Figure S2. Multivariate dissimilarity in community structure between any given two plots. We calculated dissimilarities between all plots (from the multivariate species cover data) and then separated the results according to whether or not the pair of plots was found in the same patch. Results were grouped into within-patch comparison (W) and among-patch comparison (A). We used χ^2^ distance as the dissimilarity index to compare two plots. Lower distance values indicate communities being more similar. Solid circles and error bars represent the mean and 95% confidence interval around the mean. Confidence intervals were calculated using a nonparametric bias-corrected and accelerated bootstrap (Carpenter and Bithell 2000). We found that communities in different patches were on average 18% more different than communities growing in the same patch. This estimate is obtained by averaging community dissimilarities across sampling times, which can be broken down into early succession (16% more different among-patch than within-patch), mid-succession (22%), and late succession (17%).

References

Carpenter, J., and J. Bithell. 2000. Bootstrap confidence intervals: when, which, what? A practical guide for medical statisticians. Statistics in Medicine **19**:1141-1164.
